# Optimizing dog population control strategies in Thailand using mathematical and economic modeling

**DOI:** 10.1371/journal.pntd.0013202

**Published:** 2025-07-03

**Authors:** Weerakorn Thichumpa, Anuwat Wiratsudakul, Saranath Lawpoolsri, Yanin Limpanont, Weerapong Thanapongtharm, Lauren M. Smith, Sarawut Maneewong, Wirichada Pan-ngum

**Affiliations:** 1 Department of Tropical Hygiene, Faculty of Tropical Medicine, Mahidol University, Bangkok, Thailand; 2 Mahidol Oxford Tropical Medicine Research Unit (MORU), Faculty of Tropical Medicine, Mahidol University, Bangkok, Thailand; 3 Department of Clinical Sciences and Public Health, and the Monitoring and Surveillance Center for Zoonotic Diseases in Wildlife and Exotic Animals, Faculty of Veterinary Science, Mahidol University, Nakhon Pathom, Thailand; 4 Department of Social and Environmental Medicine, Faculty of Tropical Medicine, Mahidol University, Bangkok, Thailand; 5 Department of Livestock Development, Ministry of Agriculture and Cooperatives, Bangkok, Thailand; 6 Population Health and Immunity Division, The Walter and Eliza Hall Institute of Medical Research (WEHI), Melbourne, Victoria, Australia; 7 Department of Medical Biology, University of Melbourne, Melbourne, Victoria, Australia; 8 Animal Care Center, Khao Sam Yot Town Municipality, Lopburi, Thailand; University of Abuja Faculty of Veterinary Medicine, NIGERIA

## Abstract

A mathematical model was constructed to investigate dog population dynamics and explore the impact of population management and rabies prevention. We aimed to evaluate cost-effective sterilization and vaccination strategies for dog population control and rabies prevention in Thailand. The developed compartmental model was calibrated with dog population data from Lopburi province (between 2019 and 2022) and simulated five sterilization scenarios. These measures included a combined 80% coverage of the rabies vaccine and 20% coverage of a sterilization program among non-specific dog types. Our findings indicated that sterilization programs targeting female indoor, outdoor, and stray dogs may prove to be the most effective in reducing the total dog population above 50% over a five-year period, surpassing the efficacy of the current intervention. Furthermore, the cost-effectiveness analysis showed that the two female dog sterilization strategies were cost-saving compared to the current practice, as the total costs of sterilization and vaccination decreased over time due to the reduction in the dog population. In conclusion, targeting female dog sterilization could reduce the population and was cost-saving compared to current strategies. Further data to inform dog population demographic and available resources including manpower, rabies vaccine, sterilization toolkits, and related materials will be required to fully explore intervention accessibility and feasibility within the context of rabies prevention and control in Thailand.

## Author summary

Free-roaming dogs present a risk to community health, particularly in low- and middle-income countries where dog-mediated rabies remains a critical issue. Rabies control strategies basically integrate mass vaccination with sterilization programs to prevent and control both disease transmission and population growth. However, resource constraints often limit the effectiveness of these interventions. Mathematical modeling serves as an alternative tool for assessing the potential impact of such strategies and identifying cost-effective approaches without the financial burden of real-world implementation. In this study, we developed a mathematical model to analyze dog population dynamics in Thailand, with a focus on sterilization and vaccination programs. Our findings suggest that prioritizing the sterilization of female dogs can substantially reduce the overall dog population while demonstrating greater cost-effectiveness compared to non-specific sterilization programs. This modeling approach provides critical evidence to support informed decision-making in dog population management and rabies control efforts.

## Introduction

Uncontrolled free-roaming dog populations have the potential to cause adverse impacts. These impacts encompass public health risks, socioeconomic challenges, political issues, and concerns regarding animal welfare [1–4]. Free-roaming dogs not only pose a substantial threat to human health but can also contribute to environmental degradation by introducing fecal contamination, disseminating refuse, inflicting damage to property, and creating noise pollution [5–9]. In many low- and middle-income countries, the transmission of rabies from dogs to humans is a major concern [1,9–13]. According to the World Health Organization (WHO), dogs are responsible for 99% of rabies transmissions to humans [9]. An increase in free-roaming dog populations is associated with an increasing risk of rabies outbreaks, particularly in countries where dog rabies is prevalent [10,12]. The management of dog populations is thus employed to reduce the size of free-roaming dog populations and to reduce the risk of rabies outbreaks [13]. Methods of dog population management include reproductive control (e.g., sterilization or confinement), the placing of free-roaming unowned dogs in shelters, and culling. These methods are often combined with mass rabies vaccination campaigns.

Managing the dog population is a critical factor in achieving a successful dog control program. The concept of community dogs involves dog population management programs built on existing relationships between free-roaming dogs and local inhabitants, for instance, the “Chiang Mai model” in Thailand [13]. Understanding the impact of dog population management strategies is essential to ensuring their efficiency and cost-effectiveness in controlling dog populations [13]. This requires a comprehensive understanding of dog population characteristics and demographics to target interventions appropriately. For example, understanding factors such as population size, ownership status, age and sex structures, and neutering/vaccination status is important for effective intervention planning [14]. Previous studies have aimed to survey the characteristics of dog populations across several areas globally, some of which have focused on stray (unowned) dogs [14–16], on owned dogs [17,18], or both [19]. In Thailand, the majority of free-roaming dogs are owned or are under some level of human care [13,20]. Furthermore, owned dogs are primarily responsible for rabies outbreaks in Thai settings [21].

Rabies has been listed as a notifiable disease in Thailand since 1980; a total of 370 human cases of rabies were reported that year [22]. Following this, mass vaccination campaigns and dog sterilization programs were implemented among the dog population, while post-exposure prophylaxis was administered to human individuals who had been bitten by dogs. There was a significant reduction in the number of human rabies cases between 1990 and 2020, raising the prospect of rabies elimination in Thailand [20,22,23]. Until 2013, there was a consistent correlation between the number of reported animal rabies cases and human rabies cases [23,24]. In 1995, there were 2,939 recorded animal cases of rabies, which had decreased to 1,181 cases by 2000. In 2010 and 2013, the reported numbers further declined to 249 and 117 cases, respectively [22,23]. The annual incidence of animal rabies cases reached 250 in 2014, with a further large increase to 1,476 cases in 2018, due to constraints in vaccine accessibility and policy implementation [22]. However, human rabies cases declined from 370 in 1980–215 in 2023 eventually, due to vaccination and sterilization efforts, though challenges remain in resource-limited settings [24]. This reduction reflects the demonstrable success of comprehensive rabies elimination strategies implemented to prevent the spread of the disease. While rabies vaccination has been established as a highly effective intervention in preventing fatal rabies infections in both humans and dogs [25,26], implementing vaccinations alone is likely to be insufficient to eradicate the disease. Although Thai law stipulates that dog owners must administer the rabies vaccine to their dogs annually since 1992, this requirement is not rigorously enforced, and the associated penalties are not fully implemented. Thus, the concurrent implementation of dog population control programs is considered critical for achieving and sustaining rabies eradication [27].

The World Organization for Animal Health (WOAH) advocates a multifaceted approach to eliminate dog-mediated rabies, emphasizing both mass dog vaccination campaigns (targeting at least 70% coverage) and effective dog population control measures [28]. Notably, a high-density dog population can lead to a rapid turnover of dogs, which may subsequently reduce canine vaccine coverage in endemic areas [4,29–31]. The rapid turnover of dog populations, characterized by high birth and death rates, poses a major challenge to achieving and maintaining sufficient vaccination coverage. Furthermore, as rabies is a transboundary animal disease, preventing its reintroduction from neighboring countries also requires sustained vaccination efforts [32]. Achieving optimal vaccination coverage across an entire dog population might be challenging under these conditions [30,31]. In accordance with “One Health” approaches, dog population control and management as well as mass vaccination have been prioritized as important strategies to control rabies and improve animal welfare in Thailand [9]. To effectively plan and evaluate dog population management strategies and rabies interventions, it is important to understand the ecosystem and dynamics of free-roaming dog populations. Rabies control strategies frequently aim to combine mass dog vaccination and sterilization programs, using a One Health approach. In reality, this is often challenging in terms of policy implementation, for example, due to limited materials, resources, and budgets.

Mathematical modeling helps in optimizing interventions before costly real-world implementation, particularly where data are scarce [33]. Previous studies have investigated the impact of management interventions using mathematical models to simulate dog population dynamics under different intervention scenarios [33,34]. In Thailand, estimates of dog population size and evaluation of the impact of dog population management remain scarce. Dog population management currently relies on two primary strategies implemented by government departments: mass dog vaccination and sterilization campaigns. However, the effectiveness of these methods has faced challenges due to budgetary constraints and limited resources. In this present study, we constructed a mathematical model to explore theoretical potential scenarios and provide information for policymakers to support their decision-making. We aimed to describe dog population dynamics and the impacts of dog population management strategy in Thailand. We investigated the optimal use of sterilization and vaccination programs for reducing the size of dog populations and preventing dog rabies transmission. We simulated the effects of these interventions on dog population size, as well as their cost-effectiveness. Current dog population management methods in Thailand primarily involve sterilization and some housing of dogs in shelters. Culling is not used as a method of population control due to conflicts with local religious and cultural beliefs. Under the hypothesis that female dogs may exhibit superior strategies, we compared female-focused sterilization strategies with a general sterilization program. This analysis was enriched by using a population dynamics model incorporating contextual information, aiming to inform rabies prevention and control strategies in Thailand.

## Results

### Scenario analysis

**Fig 1:**
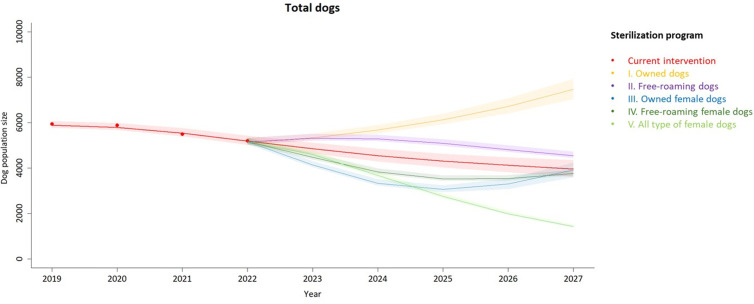
Reductions in the estimated total dog populations were targeted through sterilization programs, starting with an initial 500 dogs in 2023. This resulted in varied sterilization coverage across different types of dogs, ranging from 13% to 49%.

We calibrated the dog population dynamics model using data from Lopburi between 2019 and 2022 and predicted the number of dogs until 2027. The model well converged with the Gelman–Rubin Potential Scale Reduction Factor (PSRF) of 1.005, where the value close to 1.0 suggests that the chains have reached a stable state, and the sampling procedure is considered reliable (see S2 Text). In the subgroup analysis of model outcomes, we segmented the dog populations that had seen effective results from the sterilization program into various scenarios, including owned dogs, free-roaming dogs, and female dogs only. Compared with the current intervention, the sterilization program focusing on females showed the greatest reduction in dog population size (Fig 1).

To prioritize scenarios for sterilization programs, the model initially targeted the fixed 500 dogs for sterilization in 2023. The sterilization program targeting all types of female dogs resulted in the greatest reduction in population size, achieving over a 50% decrease by 2027, reducing the population from approximately 5,000 to lower than 2,500 dogs when compared to the current intervention. The sterilization programs for owned and free-roaming dogs did not result in reductions in dog population sizes. The scenarios involving sterilization in owned and free-roaming females reduced the dog population size over the first four years but were less effective in reducing population size compared with the current sterilization program (Fig 1).

### Cost-effectiveness analysis

**Fig 2:**
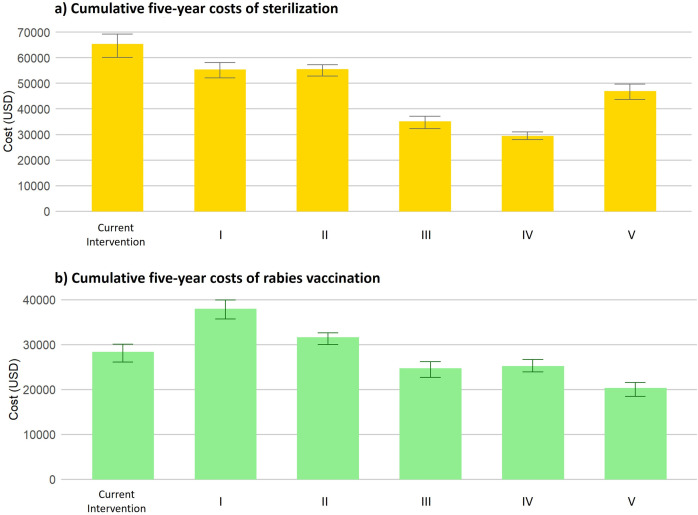
Estimated costs of dog sterilization and vaccination from 2023 to 2027. (**a**) Estimated cumulative five-year costs of sterilization. (**b**) Cumulative five-year costs of rabies vaccination at 70% coverage for the entire population. Where: the different target groups for sterilization are scenario I) Owned Dogs, II) Free-Roaming Dogs, III) Owned Female Dogs, IV) Free-Roaming Female Dogs, and V) All Female Dogs.

The cumulative costs (USD) of dog sterilization programs under various scenarios from 2023 to 2027 were estimated to be as follows. The cost of current sterilization program was USD 65.3k (95%CI 60.0-69.2k), while the cheapest program when implementing the sterilization in free-roaming female dogs (scenario IV) with USD 29.3k (95%CI 27.8-30.9k). This 5-year profit accounted for a decrease in cost by approximately 28.3% with no discount rate, and by approximately 25.3% with a 3% discount rate when compared to the current intervention cost. All other interventions showed a lower cost than the current intervention including owned female dogs (USD 35.0k, 95%CI 32.2-37.1k), all types of female dogs (USD 46.9k, 95%CI 43.6-49.6k), owned dogs (USD 55.3k, 95%CI 52.0-58.1k), free-roaming dogs (USD 55.5k, 95%CI 52.7-57.1k) (Fig 2a).

The vaccination costs (fixed at 70% coverage) varied for the different scenarios. Over five years, the cumulative cost of the current vaccination program was USD 28.4k (95% CI: 26.0–30.0k). The cheapest intervention was to sterilize all types of female dogs (scenario V) which was USD 20.3k (95%CI 18.5-21.5k). Compared to the current intervention, the reduction in the 5-year cost of rabies vaccination for the sterilization program targeting all types of female dogs was 35.2% (with no discount) and 31.5% (with 3% discount rate). Additionally, the vaccination costs for sterilization programs targeting owned female dogs (USD 24.7k, 95%CI 22.7-26.1k) and free-roaming female dogs (USD 25.2k, 95%CI 23.9-26.7k) were cheaper than the current intervention. In contrast, the vaccination costs involving the sterilization of all free-roaming dogs (USD 31.6k, 95% CI: 30.0–32.6k) or all owned dogs (USD 38.0k, 95% CI: 35.7–39.9k) was more costly (Fig 2b). Further cost analyses by strategies and by year can be found in the S3 Text.

**Fig 3:**
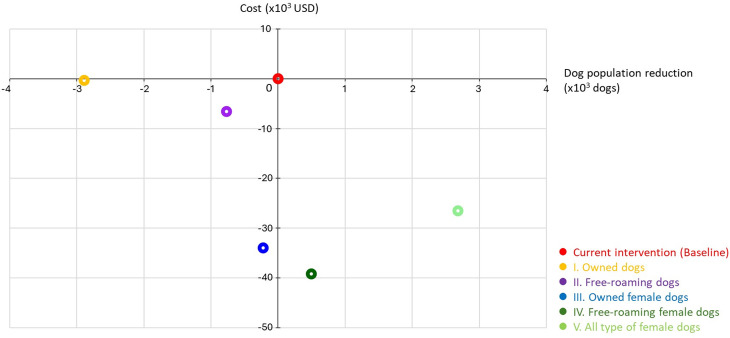
Incremental cost-effectiveness ratio (ICER) plane of five scenarios for combined sterilization and 70% coverage vaccination to control the dog population.

Our cost-effectiveness analysis indicated that combined vaccination and sterilization targeting free-roaming female (scenario IV), and all type of female dogs (scenario V) were in the cost saving quadrant of the ICER plane. Sterilizing free-roaming female dogs resulted in approximately a 28% cost reduction compared to current strategies, representing the most cost-effective approach over the five-year intervention period. While sterilizing all types of female dogs (Scenario V) was the most effective in reducing the dog population size (Fig 3).

## Discussion

The lack of comprehensive information regarding dog population dynamics and the corresponding lack of evaluations of dog population management interventions have been persistent concerns in Thailand over many years. Our study helps to address these issues as part of a broader effort to manage Thailand’s dog population and eliminate rabies. In response to the identified knowledge gap, we simulated dog-mating events to estimate annual puppy numbers, using relevant information from previous studies [35,36], and modeled the dog population dynamics in Thai context. Our model was validated against the observed data from one province in Thailand where data was available. Additionally, the model’s sensitivity analysis was performed on variations in sex and ownership type ratios, as detailed in S1 Text. Our study demonstrates that female-focused sterilization is the most cost-effective strategy for dog population control.

Our dog mortality rates estimated by the model fell in the range of estimates by the previous studies, which estimated death rates to be in the range of 0.1 to 0.7 per year [15,19,31,37,38]. However, it is crucial to note that dog mortality is influenced by various factors, including dog characteristics, study areas, and other pertinent variables (food sources, dogs’ feeders, other animals in a given area) [39,40]. Furthermore, in contexts where data on birth and death rates are limited, low-resource settings often encounter challenges such as restricted access to veterinary services or inconsistent implementation of interventions, which may result in reduced compliance rates. Similarly, areas with high population density may exhibit distinct dynamics in dog population turnover and disease transmission. Addressing these limitations is critical to ensure accurate interpretation and contextual adaptation of the model’s findings to other regions within Thailand. Additional research is necessary to address these issues and minimize potential biases.

In our study, we developed sterilization scenarios in line with the current practice where the initial number of targeted dogs was 500 per year, over a five-year plan, aligning with a period of administrative changes and policymaking decisions. Our model predictions align with findings from previous modeling studies and indicate that a sterilization program targeting female dogs can effectively reduce the overall dog population size [17,33,41]. It significantly reduces birth rates, disrupts the breeding cycle, enhances resource allocation efficiency, and provides sustainable long-term benefits for dog population management. Specifically, a sterilization program of all types of female dogs could result in a considerable decrease (>50%) in the total population over the next five years, compared with the impact of the current intervention (see S1 Table). Although the total population size in the sterilization program for owned and free-roaming female dogs was projected to eventually increase, this increase was likely attributable to the absence of sterilization efforts among the other types of female dogs. Consequently, we suggest that solely focusing on owned or free-roaming female dogs may not be sufficient for controlling population size. The critical requirement is the implementation of a sterilization program in all types of female dogs, to achieve an effective reduction in the dog population in the near future. In accordance with WHO and WOAH guidelines, Thailand’s One Health policy advocates mass dog vaccination, with an annual coverage target of 70%. The policy emphasizes a program of annual sterilization, with a particular focus on female dogs, along with mass vaccination campaigns for dogs of both sexes, deeming these interventions cost-effective [4,38,42]. Evidence from previous studies also suggests that both interventions are efficient and cost-effective strategies for reducing dog population sizes and turnover, while also mitigating rabies transmission among both dogs and humans [3,4,37,38].

Previous economic studies have demonstrated that mass dog vaccination and sterilization programs are both effective strategies for controlling rabies transmission and managing dog population size [43–45]. Although sterilization involves high initial costs, it provides long-term economic benefits by reducing the need for resources to control dog populations and decreasing the financial burden on animal shelters. The integration of both strategies offers a comprehensive and sustainable framework for dog population management and rabies control. Based on this evidence, we considered five scenarios projecting reductions in dog populations over the next five years (2023–2027), comparing to the current intervention. We conducted an economic analysis to assess the cost-effectiveness of the proposed dog population control and rabies prevention strategies. We incorporated the cost of interventions in the year 2027, visualizing the results in the ICER plane (Fig 3). Compared with the current intervention, our cost-effectiveness analysis indicated that interventions focused on all types of female dogs were most effective for dog population control. Despite the cost of sterilizing all females not being the lowest, the costs of vaccinating all females were the most economical, achieving cost reductions in the future (see S3 Text). It is important to note that the cost of interventions probably vary across different regions due to factors such as endemic areas, local budgets and resources, policy implementation, transportation, and Government and NGOs support.

This study had some limitations, primarily arising from the lack of comprehensive data, which posed a considerable challenge in understanding dog population dynamics within the context of rabies control. Successful rabies control programs typically integrate a variety of strategies, such as mass dog vaccination, sterilization campaigns, dog population management, and public awareness efforts. However, the effectiveness of these initiatives is contingent on accurate and up-to-date data on dog populations. Incomplete coverage of sterilization and vaccination, potential inaccuracies in population estimates, and varying levels of community compliance could influence the effectiveness of intervention strategies. Additionally, the assumption of area specific fixed death rates and constant sterilization effectiveness simplifies projections may not accurately reflect real-world variability. Uncertainty in birth and death rates and regional variability may also affect generalizability. Future research should refine these estimates by incorporating dynamic parameters, such as seasonally varying mortality rates or sterilization effectiveness influenced by age and behavioral factors. The variability in data quality, along with limited or unreliable data in local, provincial, and regional levels such as birth and death rates, vaccination and sterilization coverage, and compliance levels, highlights discrepancies in accuracy, completeness, and consistency, all of which can significantly impact the model outcomes. Given that such data is not available across all settings, along with regional variations in dog population characteristics, model adjustments will be necessary prior to its application in different areas and contexts. The regional factors can also influence both the baseline population dynamics and the effectiveness of interventions, underscoring the need for localized calibration of the model. Also, in this study we did not account for the carrying capacity of a given area for the local dog population, which could potentially influence long-term model predictions.

Our intervention scenarios did not include a culling program, due to cultural beliefs and religious concerns in Thailand regarding killing animals. Putting dogs in shelters and the immigration of dogs were also excluded from the population dynamics model due to their unique characteristics and because of insufficient information. Future model developments should consider these dog population characteristics, as well as exploring aspects such as abandonment and adoption to explore additional impacts on dog population dynamics. Despite limitations in the available dog population data, our study has introduced an alternative modeling approach that can offer valuable insights to support policymaking decisions and could be beneficial for rabies prevention and control.

Limited resources, including budgets, materials, manpower, staff, and transportation, may constrain the ability to conduct widespread intervention campaigns. Managing dog populations is complex, involving multiple aspects. Notably, in reality the sterilization of dogs of a specific sex may be challenging. When dog catchers are catching dogs, they may be unable to determine their sex, and they may feel that it is better to simply sterilize any dog that they catch. It is generally more complex and time-consuming to sterilize female dogs than male dogs. This is primarily because spaying involves abdominal surgery to remove the ovaries and often the uterus, while sterilization in male dogs, commonly performed in sterilization campaigns via gonadectomy, is a less invasive procedure that involves the removal of the testicles [46]. Additionally, catching free-roaming dogs to sterilize them can be more challenging compared with catching owned dogs. Free-roaming dogs are not directly controlled and are by definition allowed to roam freely, making it difficult to catch them for sterilization. Owned dogs, on the other hand, are generally more accessible because their owners can bring them in directly for the procedure. However, if a program is effective and the size of the dog population is decreasing, catching dogs will become more difficult, due to their smaller numbers. In addition, public perception plays a crucial role, as community acceptance of sterilization, particularly female-focused programs, may vary due to cultural beliefs and concerns about animal welfare. Furthermore, logistical difficulties in rural areas, including limited veterinary services, resource constraints, and difficulties in accessing remote communities, can hinder the effectiveness of large-scale interventions. Therefore, it is crucial that any decisions regarding sterilization and the selection of the appropriate methods should only be made after careful consideration. This decision may be influenced by a variety of factors, including a dog’s health, age, and type, as well as accessibility and the feasibility of implementing intervention campaigns based on the resources available. Acknowledging these barriers provides a more balanced perspective on the feasibility of implementing such interventions on a larger scale and highlights the need for adaptive strategies to improve their long-term sustainability.

Despite these challenges, we anticipate that the results of this study can provide valuable information to inform policymaking decisions aimed at controlling both dog populations and rabies transmission in Thailand and similar contexts. In addition, substantial policy changes that adjust a country’s operational rules may impact on the effectiveness of public health initiatives and the overall success of rabies control programs. Changes in administrations and policies might affect the design and implementation of mass dog vaccination and sterilization programs. The success of rabies elimination efforts relies heavily on a high level of intervention coverage and good management. Policy adjustments should be focused on ensuring continued or enhanced support for intervention campaigns, as well as accessibility, outreach, and community engagement [9]. Also, prioritizing the development and maintenance of robust data systems is necessary to inform evidence-based decision-making, resource allocation, and evaluation of dog population control and rabies elimination programs. In future studies, we will focus on applying our dog population dynamics model to other geographical areas and at the national level, as well as abandonment and adoption. We recommend integrating female sterilization into national rabies programs, with pilot studies to assess feasibility. Additionally, future research should integrate models of human dog bites and rabies cases to estimate the disease burden in humans. This approach would enable the assessment of the cost-effectiveness of vaccination and dog population management (DPM) programs in alignment with WHO guidelines. Accurate disease burden estimation is crucial for designing targeted interventions and optimizing resource allocation, ultimately enhancing rabies control and prevention efforts.

## Methods

### Dog population surveys

Common methods for estimating dog population size include observational, sight-resight, and mark-recapture surveys [14–19,47]. Based on previous studies, we structured our dog population dynamics model using data from household surveys. The survey study collected data with monthly updates from dog owners across four regions of Thailand (Central, Northern, Northeastern, and Southern) between 2019 and 2021 [48]. Briefly, this investigation surveyed dog populations to characterize subpopulations and their inherent characteristics that we could then incorporate in our model. Based on this work, we established a model framework to distinguish three primary types of dog population within Thai communities: indoor, outdoor, and stray. We defined “indoor dogs” as confined, owned dogs; “outdoor dogs” as unconfined, owned dogs; and “stray dogs” as free-roaming, unowned dogs. Notably, for our scenario analysis, we further grouped dog populations as “confined dogs” and “free-roaming dogs” (outdoor and stray) to investigate broader population dynamics.

**Fig 4:**
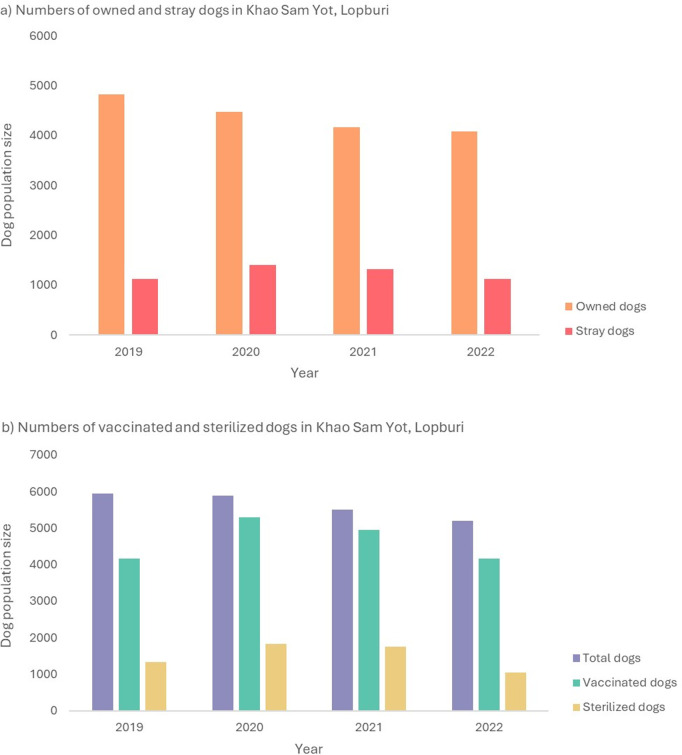
Reported dog population by a survey conducted between 2019 and 2022 in Khao Sam Yot, Mueang Lopburi district, Lopburi province. (**a**) Numbers of owned and stray dogs. (**b**) Number of vaccinated and sterilized dogs.

In Thailand, comprehensive household surveys of the dog population, including records of sterilized and vaccinated dogs, remain scarce. Additionally, the accuracy and validity of the available data are also limited. A four-year data collection effort was conducted in Khao Sam Yot, Mueang Lopburi District, Lopburi Province, using a specific method with follow-up procedures based on household and door-to-door surveys. Khao Sam Yot covers a total area of approximately 32.5 km² with the population of 30,129 [49,50]. To construct and calibrate the model, we used household survey data from Lopburi province (between 2019 and 2022), where dog-to-human ratios averaged 1:5.3. The recorded total dog populations were 5,943, 5,885, 5,493, and 5,202 dogs, respectively. This area had achieved a substantial level of coverage of both mass dog vaccination and dog sterilization (Fig 4) [51]. We considered to use dog population data obtained from this area to calibrate our model for the “current intervention” scenario (i.e., it combined 80% coverage of the rabies vaccine and 20% coverage of the sterilization program among non-specific dog types).

### Dog population structure and model description

**Fig 5:**
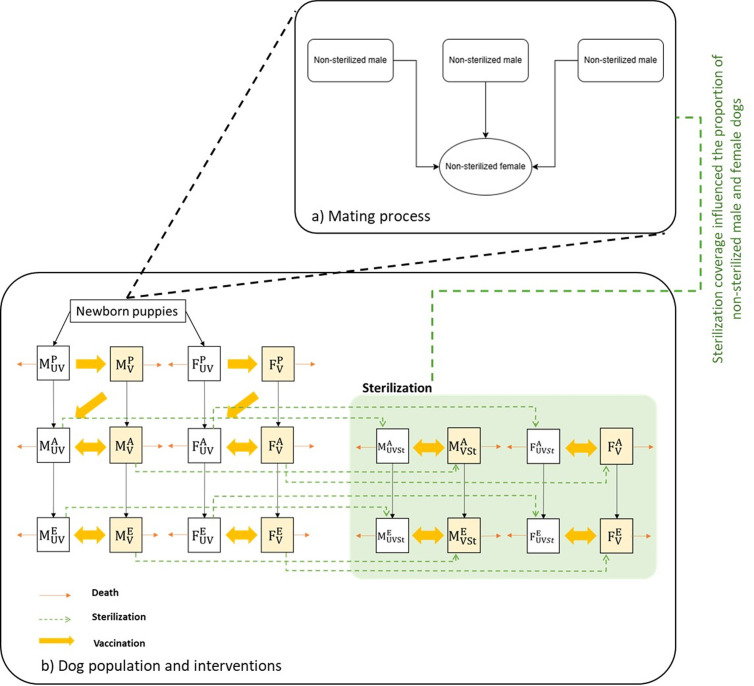
Model structure. (**a**) Schematic concept of mating process. (**b**) Dog population model and interventions for each type of dog (i.e., indoor, outdoor, or stray dogs). Where: M*,* male dog; F, female dog; P, puppy*;* A, adult*;* E*,* elderly; UV, unvaccinated; V, vaccinated; St, sterilized.

We structured the model by dog type (indoor, outdoor, stray), sex, and age (puppy, adult, elderly) to simulate dog population dynamics. These were further subdivided into confined and free-roaming dogs. The ratio of owned to stray dogs was approximately 4.2:1 [20,51]. We applied a set of ordinary differential equations to describe the rates of change of dog population dynamics (see S1 Text). Based on the dog population categories, equations for the three main types of dogs (indoor, outdoor, stray) were constructed, using the same pattern of model structure (Fig 5). All types of dog population dynamics were started with the initial number of newborn puppies in the puppy stage. For new population inputs, the estimated number of puppies from the mating simulation (Fig 5a) was multiplied by the number of female dogs in each type of population before flowing into the dynamics model. Given the male to female ratio of 1.3:1 [20], relative numbers of male and female puppies flowed to the sexual stage (adult) after six months and entered the older stage after eight years. We assumed that dogs lived for a maximum of 13 years, therefore dogs were removed from our model after the age of 13 [52]. Vaccination was applied to all types of dogs. Maternal antibodies were not considered, and the length of vaccine protection was assumed to be 1 year. While sterilization program was applied to adult and elderly dogs only. Key parameters were incorporated into the dynamics model, including newborns, death rate, vaccination coverage, and sterilization coverage (see S1 Text).

### Simulation of dog reproductive rate

We considered the reproductive patterns of dogs to determine the probability of mating and the occurrence of newborn puppies. Female dogs can be in estrous once or twice a year, based on their biological reproductive cycle (six- or seven-month cycle) [53], whereas male dogs can mate throughout the year depending on females allowing them to do so [53–55]. Thus, dogs are capable of reproducing throughout the year, i.e., non-seasonal breeding [35,36,53,56], although some studies have reported that seasonality may affect the reproductive cycle of female dogs [54,55]. However, it is the case that dogs’ breeding can be altered by various factors, such as nutrition, temperature, environment, and biological characteristics [35,54,56]. In this study, we assumed that dogs could reproduce once a year. We also considered that the birth rate of newborn puppies could vary based on the mating process, which was influenced by age and sex ratios, mating frequency, and sterilization status. In the case of indoor dogs, as they were confined, we reduced the number of indoor newborn dogs by approximately 23% [3,57].

We simulated the mating processes using dog status derived from the dog population dynamics model. The mating process was simulated independently from the population dynamics model. Briefly, we assumed non-seasonal breeding, with female dogs mating ≥3 times annually to produce an average litter of six puppies. For the details of mating process, we assumed: i) both female and male dogs could mate during the sexual stage (mature), and ii) for any female dogs, provided they mated ≥3 times per year with any male dogs, this would result in whelping. In the initial phase, we established a population of 10,000 dogs encompassing both sexes, assuming that 80% of the dogs were on the sexual stage. These values were derived from the survey we conducted between 2019 and 2021. The number of mating times per female dog was sampled from a uniform distribution, ranging between 0 and 10 times (informed by the dog population survey and expert opinion). For female dogs whose sampled mating number was ≥ 3 times, this would result in whelping [58]. The number of newborn puppies per female dog was sampled from a Poisson distribution, with a mean of six puppies per female [35,36]. The proportion of indoor dogs available for mating was reduced by 23% based on the impact of dog confinement [3,57,59]. After simulation, which considered factors that could affect the reproductive rate (i.e., litter size, number of mating times, sterilization coverage, and proportion of dogs in the sexual stage), the estimated numbers of newborn puppies were input and then multiplied by the numbers of female dogs in the population model annually.

### Model calibration

We calibrated our model using data on the size of the dog population in Lopburi province between 2019 and 2022. A sterilization campaign was introduced in this area in 2019 and ran for the duration of this period. As detailed data on the characteristics of this dog population were not available, we calibrated the total size of the dog population (i.e., the sum of all compartments) to estimates of the dog population size in Lopburi province. The compartmental model was solved using the ordinary differential equations (ODE) function in R (version 4.3.2) [60]. Model fitting was carried out using the Markov chain Monte Carlo (MCMC) method as versatile and robust approach, implemented with the Bayesian Tools R package [61]. The population dynamics model was run and fitted to the total population size between 2019 and 2022. In the model fitting, the number of iterations was 10,000 and burn-in of 500 (see S2 Text). The convergence was assessed by several measures, Gelman–Rubin statistics for convergence [62,63] and the target acceptance rates [64]. We estimated death rates for the dog population, i.e., free-roaming puppy death rate and free-roaming adult and elderly death rate (see S1 Text).

### Scenario analysis

The dynamics model simulated the total number of dogs under the current intervention in Khao Sam Yot. The study primarily aimed to predict the total dog population at specific time points. Focusing on the dog sterilization program, and randomly targeting dogs of each type, we investigated five sterilization scenarios, which were: I, owned dogs; II, free-roaming dogs; III, owned female dogs; IV; free-roaming female dogs; and V, all types of female dogs. We used our model to forecast outcomes over a five-year period, taking into account administrative shifts and policy formulation processes.

The main scenarios were prioritized based on sterilization programs of dogs in each type (Table 1). These scenarios included sterilization targeting owned dogs, free-roaming dogs, owned female dogs, free-roaming female dogs, and all types of female dogs*.* Notably, in Thailand, policymakers and non-governmental organizations (NGOs) overseeing dog population management strategize interventions with a focus on addressing two key questions: “Given limited budgets and the need for cost-effective sterilization programs, which type of dogs should be the priority?” and “How many dogs must be sterilized to effectively control population growth in the coming years?” As part of this approach, during the initial year of the model’s prediction, we implemented sterilization programs targeting an initial cohort of 500 dogs starting in the year 2023. We evaluated the impact of the sterilization program on dog population size, while rabies vaccination cost was included in the rabies prevention package together with sterilization subject to the economic analysis. The model outcomes served as valuable support for policymakers in making well-informed decisions.

**Table 1:** Scenario analysis of alternative sterilization programs.

Scenario	Description*	Estimated number of dogs of each type**	Coverage of the dog type given the fixed target of initial 500 sterilized dogs
I. Owned dogs	Sterilization of owned dogs (random between indoor and outdoor dogs)	3,900	13%
II. Free-roaming dogs	Sterilization of free-roaming dogs (random between outdoor and stray dogs)	2,700	19%
III. Owned female dogs	Sterilization of owned female dogs (random between female indoor and female outdoor dogs)	1,430	35%
IV. Free-roaming female dogs	Sterilization of free-roaming female dogs (random between female outdoor and female stray dogs)	1,020	49%
V. All types of female dogs	Sterilization of all types of female dogs (random female indoor, female outdoor, and female stray dogs)	2,000	25%

*Proportionally random sampling from target groups in each scenario.

** The total number of dogs was approximately 5,200 in 2022. Given limited data, the number of dogs in each type were calculated from the model’s prediction based on previous studies [20,51].

### Cost consideration and cost-effectiveness analysis

All costs were considered based on the model’s predictions for the upcoming five years (2023–2027), using data from Lopburi province. Costs were also discounted at 3% annually for future values. The cost associated with each intervention was calculated by determining the average expenditure per dog, in US dollars (1 USD = 35 THB in 2024). For dog sterilization, the average costs per dog were estimated to be 12 USD for castration, 23 USD for spaying, and an additional 5 USD allocated for anesthesia in cases involving uncatchable dogs [24,51]. For rabies vaccination, the established cost was set at 1.5 USD per dog per year [24,51]. These costs represent the average prices established by local government regulations, considering the essential economic aspects, including time consumption, transportation, human resources, and associated expenses [51]. To assess the cost-effectiveness of scenarios in comparison to the current intervention (Year 2027), we determined the incremental cost-effectiveness ratio (ICER). The ICER was calculated by analyzing the incremental costs for the sterilization and vaccination package for the 5-year timeline of each intervention compared with the current practice versus the benefit in terms of dog population reduction from applying the intervention as opposed to the current practice (see S3 Text). When the net present value was estimated, a 3% discount rate per year was incorporated into the calculation.

### Estimating rabies vaccine coverage for cost considerations

We calculated the effective reproductive number (R_t_) using the equation, $R_{t}=\;R_{0}\times (\frac{S}{N}$), where R₀ was taken from literature (2.44) [11], S was the number of unvaccinated dogs at time t, and N was the total dog population at time t. If R_t_ is less than 1, it indicates the likelihood of a decline in disease cases, in this case indicating that rabies is under control. In our estimation, R_t_ was less than 1 when vaccination coverage reached ≥60% (R_t _= 0.98) (see S3 Text). A 70% vaccination coverage was used for the economic analysis, based on WHO and WOAH guidelines. This implied that controlling the spread of rabies could be achieved by vaccinating a sufficient percentage of dogs. Such estimates were crucial for guiding vaccination efforts, especially when considering cost implications.

## Supporting information

S1 Text Model details and outputs. (DOCX)

S2 Text Model fitting and related information. (DOCX)

S3 Text Cost evaluation and related information. (DOCX)

S1 Table The estimated number of dogs. (DOCX)
