# Supplementary material for: Optimizing dog population control strategies in Thailand using mathematical and economic modeling
Source: PLoS Negl Trop Dis. 2025 Jul 3;19(7):e0013202. doi: 10.1371/journal.pntd.0013202 (PMC12225835; doi:10.1371/journal.pntd.0013202)
Supplement: S1 Text — (DOCX) [file pntd.0013202.s001.docx]

**Optimizing dog population control strategies in Thailand using mathematical and economic modeling**

**Supporting Information S1 Text: Model details and outputs**

# **1. Abbreviations used in dog compartments**

| **Abbreviation** | **Description** |
| --- | --- |
| I | Indoor |
| O | Outdoor |
| S | Stray |
| F | Female |
| M | Male |
| Pup | Puppy |
| A | Adult |
| E | Older (Elderly) |
| UV | Unvaccinated dog |
| V | Vaccinated dog |

# **2. Model parameters**

| **Parameter** | **Description** | **Value** | **Unit** | **Reference** |
| --- | --- | --- | --- | --- |
| newborn | Indoor newborns per female dog | 1.40-1.90 | Per year | Simulation |
|  | Outdoor newborns per female dog | 1.80–2.40 | Per year |  |
|  | Stray newborns per female dog | 1.80–2.40 | Per year |  |
| D | Indoor death rate | 0.19 | Per year | Assumption |
|  | Free-roaming puppy death rate | 0.33-0.42 | Per year | Fitting |
|  | Free-roaming adult and elderly death rate | 0.33-0.36 | Per year | Fitting, [1] |
| m | Multiplier for correction of aging processes | 5 | - | Calculation |
| vm | Multiplier for correction of vaccine waning process | 5 | - | Calculation |
| r | Female to male fraction | 0.43 | - | Survey |
| freq.mate | Frequency of mating | Uniform (0, 10) | Per year | Assumption |
| λ | Average litter size | 6 | Per year | [2, 3] |
| St | Sterilization coverage | 0.20 | Per year | [4] |
| vc | Vaccination coverage | 0.80 | Per year | [4] |

For Lopburi province, the sterilization (St) of all indoor and outdoor dogs was fixed at 20%, while for the stray dogs was 10%. The vaccination coverage (vc) was fixed at 80%.

# **3. Overview of dog population model equations** (Unit of time: day)

*The generic set of ODEs*

$$\frac{d\mathrm{Dog}_{(no intervention)}}{\mathrm{dt}}= Birth - Aging - Death - Vaccine - Sterilization + Vaccine waning$$

$$\frac{d\mathrm{Dog}_{V}}{\mathrm{dt}}= Vaccine - Aging - Death - Sterilization - Vaccine waning$$

$$\frac{d\mathrm{Dog}_{St}}{\mathrm{dt}}= Sterilization - Aging - Death - Vaccine+{Vaccine waning}_{St}$$

$$\frac{d\mathrm{Dog}_{StV}}{\mathrm{dt}}= Sterilization - Aging - Death+Vaccine - {Vaccine waning}_{St}$$

Where:

Birth = newborn x F

Death = Death rate x Dog_(each compartment)_

Aging = the aging process starts from puppy stage (6 months, i.e. 183 days) to adult (until 8 years, i.e. 365x7.5) to elder stages (8+ years)

Sterilization = the sterilization coverage x Dog_(each compartment)_

Vaccine = rabies vaccine coverage x Dog_(each compartment)_

Vaccine waning = the vaccine waning from vaccination stage of non-sterilized dog_(each compartment)_

Vaccine waning_st_ = the vaccine waning from vaccination stage of sterilized dog_(each compartment)_

# 3.1 ODEs for population dynamics

**Female dogs**

$$\frac{dF^{\mathrm{Pup}}}{\mathrm{dt}}=\left( r\times\left( \frac{\mathrm{newborn}}{365} \right)\times\left( F_{\mathrm{UV}}^{A}+F_{\mathrm{UV}}^{E}+F_{V}^{A}+F_{V}^{A} \right) \right)-\left( m\times\left( \frac{1}{183} \right)\times F_{\mathrm{UV}}^{A} \right)-\left( D\times F_{\mathrm{UV}}^{\mathrm{Pup}} \right)-(\mathrm{vc}\times F_{\mathrm{UV}}^{\mathrm{Pup}})$$

$$\frac{dF^{A}}{\mathrm{dt}}=\left( m\times\left( \frac{1}{183} \right)\times F_{\mathrm{UV}}^{A} \right)-\left( m\times\left( \frac{1}{\left( 365\times7.5 \right)} \right)\times F_{\mathrm{UV}}^{A} \right)-\left( D\times F_{\mathrm{UV}}^{A} \right)-\left( \mathrm{vc}\times F_{\mathrm{UV}}^{A} \right)-\left( St\times F_{\mathrm{UV}}^{A} \right)+\left( vm\times{(\frac{1}{365})\times F}_{V}^{A} \right)$$

$$\frac{dF^{E}}{\mathrm{dt}}=\left( m\times\left( \frac{1}{\left( 365\times7.5 \right)} \right)\times F_{\mathrm{UV}}^{A} \right)-\left( m\times\left( \frac{1}{\left( 365\times5 \right)} \right)\times F_{\mathrm{UV}}^{E} \right)-\left( D\times F_{\mathrm{UV}}^{E} \right)-\left( \mathrm{vc}\times F_{\mathrm{UV}}^{E} \right)-(St\times F_{\mathrm{UV}}^{E})+\left( vm\times{(\frac{1}{365})\times F}_{V}^{E} \right)$$

**Male dogs**

$$\frac{dM^{\mathrm{Pup}}}{\mathrm{dt}}=\left( (1-r)\times\left( \frac{\mathrm{newborn}}{365} \right)\times\left( F_{\mathrm{UV}}^{A}+F_{\mathrm{UV}}^{E}+F_{V}^{A}+F_{V}^{A} \right) \right)-\left( m\times\left( \frac{1}{183} \right)\times M_{\mathrm{UV}}^{A} \right)-\left( D\times M_{\mathrm{UV}}^{\mathrm{Pup}} \right)-(\mathrm{vc}\times M_{\mathrm{UV}}^{\mathrm{Pup}})$$

$$\frac{dM^{A}}{\mathrm{dt}}=\left( m\times\left( \frac{1}{183} \right)\times M_{\mathrm{UV}}^{A} \right)-\left( m\times\left( \frac{1}{\left( 365\times7.5 \right)} \right)\times M_{\mathrm{UV}}^{A} \right)-\left( D\times M_{\mathrm{UV}}^{A} \right)-\left( \mathrm{vc}\times M_{\mathrm{UV}}^{A} \right)-\left( St\times M_{\mathrm{UV}}^{A} \right)+\left( vm\times{(\frac{1}{365})\times M}_{V}^{A} \right)$$

$$\frac{dM^{E}}{\mathrm{dt}}=\left( m\times\left( \frac{1}{\left( 365\times7.5 \right)} \right)\times M_{\mathrm{UV}}^{A} \right)-\left( m\times\left( \frac{1}{\left( \left( 365\times5 \right) \right)} \right)\times M_{\mathrm{UV}}^{E} \right)-\left( D\times M_{\mathrm{UV}}^{E} \right)-\left( \mathrm{vc}\times M_{\mathrm{UV}}^{E} \right)-(St\times M_{\mathrm{UV}}^{E})+\left( vm\times{(\frac{1}{365})\times M}_{V}^{E} \right)$$

# 3.2 ODEs for sterilization program

**Female dogs**

$$\frac{dF_{\mathrm{St}}^{A}}{\mathrm{dt}}=\left( St\times F_{\mathrm{UV}}^{A} \right)-\left( m\times\left( \frac{1}{\left( 365\times7.5 \right)} \right)\times F_{\mathrm{St}}^{A} \right)-\left( D\times F_{\mathrm{St}}^{A} \right)-\left( \mathrm{vc}\times F_{\mathrm{St}}^{A} \right)+\left( vm\times{(\frac{1}{365})\times F}_{\mathrm{StV}}^{A} \right)$$

$$\frac{dF_{\mathrm{St}}^{E}}{\mathrm{dt}}=\left( St\times F_{\mathrm{UV}}^{E} \right)+\left( m\times\left( \frac{1}{\left( 365\times7.5 \right)} \right)\times F_{\mathrm{St}}^{A} \right)-\left( D\times F_{\mathrm{St}}^{E} \right)-\left( \mathrm{vc}\times F_{\mathrm{St}}^{E} \right)+\left( vm\times{(\frac{1}{365})\times F}_{\mathrm{StV}}^{E} \right)-\left( m\times\left( \frac{1}{\left( 365\times5 \right)} \right)\times F_{\mathrm{St}}^{E} \right)$$

**Male dogs**

$$\frac{dM_{\mathrm{St}}^{A}}{\mathrm{dt}}=\left( St\times M_{\mathrm{UV}}^{A} \right)-\left( m\times\left( \frac{1}{\left( 365\times7.5 \right)} \right)\times M_{\mathrm{St}}^{A} \right)-\left( D\times M_{\mathrm{St}}^{A} \right)-\left( \mathrm{vc}\times M_{\mathrm{St}}^{A} \right)+\left( vm\times{(\frac{1}{365})\times M}_{\mathrm{StV}}^{A} \right)$$

$$\frac{dM_{\mathrm{St}}^{E}}{\mathrm{dt}}=\left( St\times M_{\mathrm{UV}}^{E} \right)+\left( m\times\left( \frac{1}{\left( 365\times7.5 \right)} \right)\times M_{\mathrm{St}}^{A} \right)-\left( D\times M_{\mathrm{St}}^{E} \right)-\left( \mathrm{vc}\times M_{\mathrm{St}}^{E} \right)+\left( vm\times{(\frac{1}{365})\times M}_{\mathrm{StV}}^{E} \right)-\left( m\times\left( \frac{1}{\left( 365\times5 \right)} \right)\times M_{\mathrm{St}}^{E} \right)$$

# 3.3 ODEs for vaccination program

**Female dogs**

$$\frac{dF_{V}^{\mathrm{Pup}}}{\mathrm{dt}}=\left( \mathrm{vc}\times F_{\mathrm{UV}}^{\mathrm{Pup}} \right)-\left( D\times F_{V}^{\mathrm{Pup}} \right)-\left( m\times\left( \frac{1}{183} \right)\times F_{V}^{\mathrm{Pup}} \right)$$

$$\frac{dF_{V}^{A}}{\mathrm{dt}}=\left( \mathrm{vc}\times F_{\mathrm{UV}}^{\mathrm{Pup}} \right)-\left( vm\times\left( \frac{1}{\left( 365 \right)} \right)\times F_{V}^{A} \right)-\left( D\times F_{V}^{A} \right)-\left( St\times F_{V}^{A} \right)+\left( m\times\left( \frac{1}{183} \right)\times F_{V}^{\mathrm{Pup}} \right)-\left( m\times{(\frac{1}{365\times7.5})\times F}_{V}^{A} \right)$$

$$\frac{dF_{V}^{E}}{\mathrm{dt}}=\left( \mathrm{vc}\times F_{\mathrm{UV}}^{E} \right)-\left( vm\times\left( \frac{1}{\left( 365 \right)} \right)\times F_{V}^{E} \right)-\left( D\times F_{\mathrm{UV}}^{E} \right)-\left( St\times F_{V}^{E} \right)+\left( m\times\left( \frac{1}{\left( 365*7.5 \right)} \right)\times F_{V}^{A} \right)-\left( m\times\left( \frac{1}{\left( 365*5 \right)} \right)\times F_{V}^{E} \right)$$

**Male dogs**

$$\frac{dM_{V}^{\mathrm{Pup}}}{\mathrm{dt}}=\left( \mathrm{vc}\times M_{\mathrm{UV}}^{\mathrm{Pup}} \right)-\left( D\times M_{V}^{\mathrm{Pup}} \right)-\left( m\times\left( \frac{1}{183} \right)\times M_{V}^{\mathrm{Pup}} \right)$$

$$\frac{dM_{V}^{A}}{\mathrm{dt}}=\left( \mathrm{vc}\times M_{\mathrm{UV}}^{\mathrm{Pup}} \right)-\left( vm\times\left( \frac{1}{\left( 365 \right)} \right)\times M_{V}^{A} \right)-\left( D\times M_{V}^{A} \right)-\left( St\times M_{V}^{A} \right)+\left( m\times\left( \frac{1}{183} \right)\times M_{V}^{\mathrm{Pup}} \right)-\left( m\times{(\frac{1}{365\times7.5})\times M}_{V}^{A} \right)$$

$$\frac{dM_{V}^{E}}{\mathrm{dt}}=\left( \mathrm{vc}\times M_{\mathrm{UV}}^{E} \right)-\left( vm\times\left( \frac{1}{\left( 365 \right)} \right)\times M_{V}^{E} \right)-\left( D\times M_{\mathrm{UV}}^{E} \right)-\left( St\times M_{V}^{E} \right)+\left( m\times\left( \frac{1}{\left( 365*7.5 \right)} \right)\times M_{V}^{A} \right)-\left( m\times\left( \frac{1}{\left( 365*5 \right)} \right)\times M_{V}^{E} \right)$$

# 3.4 ODEs for combination of Sterilization and Vaccination

**Female dogs**

$$\frac{dF_{\mathrm{StV}}^{A}}{\mathrm{dt}}=\left( \mathrm{vc}\times F_{\mathrm{St}}^{A} \right)+\left( St\times F_{V}^{A} \right)-\left( vm\times{(\frac{1}{365})\times F}_{\mathrm{StV}}^{A} \right)-\left( m\times\left( \frac{1}{\left( 365\times7.5 \right)} \right)\times F_{\mathrm{StV}}^{A} \right)-\left( D\times F_{\mathrm{StV}}^{A} \right)$$

$$\frac{dF_{\mathrm{StV}}^{E}}{\mathrm{dt}}=\left( \mathrm{vc}\times F_{\mathrm{St}}^{E} \right)+\left( St\times F_{V}^{E} \right)-\left( vm\times{\left( \frac{1}{365} \right)\times F}_{\mathrm{StV}}^{E} \right)+\left( m\times\left( \frac{1}{\left( 365\times7.5 \right)} \right)\times F_{\mathrm{StV}}^{E} \right)-\left( m\times\left( \frac{1}{\left( 365\times5 \right)} \right)\times F_{\mathrm{StV}}^{E} \right)-\left( D\times F_{\mathrm{StV}}^{A} \right)$$

**Male dogs**

$$\frac{dM_{\mathrm{StV}}^{A}}{\mathrm{dt}}=\left( \mathrm{vc}\times M_{\mathrm{St}}^{A} \right)+\left( St\times M_{V}^{A} \right)-\left( vm\times{(\frac{1}{365})\times M}_{\mathrm{StV}}^{A} \right)-\left( m\times\left( \frac{1}{\left( 365\times7.5 \right)} \right)\times M_{\mathrm{StV}}^{A} \right)-\left( D\times M_{\mathrm{StV}}^{A} \right)$$

$$\frac{dM_{\mathrm{StV}}^{E}}{\mathrm{dt}}=\left( \mathrm{vc}\times M_{\mathrm{St}}^{E} \right)+\left( St\times M_{V}^{E} \right)-\left( vm\times{\left( \frac{1}{365} \right)\times M}_{\mathrm{StV}}^{E} \right)+\left( m\times\left( \frac{1}{\left( 365\times7.5 \right)} \right)\times M_{\mathrm{StV}}^{E} \right)-\left( m\times\left( \frac{1}{\left( 365\times5 \right)} \right)\times M_{\mathrm{StV}}^{E} \right)-\left( D\times M_{\mathrm{StV}}^{A} \right)$$

Note: that D, the death rate in the ODEs varied between indoor and free-roaming, as well as puppies and adult/elderly (see **Model parameters** above)

# **4. Estimated birth rates from dog population dynamics model.**

|  | **Birth rate** | | | | |
| --- | --- | --- | --- | --- | --- |
| **Scenario of sterilization** | **Year 2023** | **Year 2024** | **Year 2025** | **Year 2026** | **Year 2027** |
| Current intervention | 0.04 | 0.05 | 0.05 | 0.05 | 0.05 |
| Owned dogs | 0.07 | 0.06 | 0.06 | 0.06 | 0.06 |
| Free-roaming dogs | 0.06 | 0.06 | 0.06 | 0.06 | 0.07 |
| Owned female dogs | 0.05 | 0.06 | 0.07 | 0.08 | 0.09 |
| Free-roaming female dogs | 0.04 | 0.05 | 0.06 | 0.06 | 0.07 |
| All type of female dogs | 0.03 | 0.02 | 0.02 | 0.02 | 0.01 |

# **5. Sensitivity analysis**

The sensitivity analysis was conducted using the optimal scenario of sterilizing all type of female dogs (the best scenario), with variations in both sex and ownership type ratios. These results were compared the total number of dogs in 2027 against the baseline scenario (male-to-female ratio of 1.3:1 and owned-to-stray dog ratio of 80:20). At the baseline male-to-female ratio of 1.3:1, variations in the owned-to-stray dog ratio resulted in only minor changes (a maximum of 7.4%) in the total dog population. The trend of dog population size was similar in all analyses. (See Table and Figure below)

5.1) The estimated number of dogs in 2027 from sensitivity analysis

|  | Male-to-Female ratio | | | |
| --- | --- | --- | --- | --- |
| Owned-to-Stray ratio | 1.4:1 | 1.3:1 | 1.2:1 | 1:1 |
| 20:80 | 664 | 756 | 828 | 1087 |
| 50:50 | 644 | 711 | 803 | 1033 |
| 80:20 | 636 | 704* | 761 | 1009 |

**The number of dogs from baseline scenario*

5.2) The estimated number of dogs in 2027 from sensitivity analysis

# **References**

1. Komol P, Sommanosak S, Jaroensrisuwat P, Wiratsudakul A, Leelahapongsathon K. The Spread of Rabies Among Dogs in Pranburi District, Thailand: A Metapopulation Modeling Approach. Front Vet Sci. 2020;7:570504. Epub 2020/12/18. doi: 10.3389/fvets.2020.570504. PubMed PMID: 33330692; PubMed Central PMCID: PMCPMC7710610.

2. Chatdarong K, Tummaruk P, Sirivaidyapong S, Raksil S. Seasonal and breed effects on reproductive parameters in bitches in the tropics: a retrospective study. J Small Anim Pract. 2007;48(8):444-8. Epub 2007/06/15. doi: 10.1111/j.1748-5827.2007.00342.x. PubMed PMID: 17559524.

3. Wigham EE, Moxon RS, England GCW, Wood JLN, Morters MK. Seasonality in oestrus and litter size in an assistance dog breeding colony in the United Kingdom. Vet Rec. 2017;181(14):371. Epub 2017/09/04. doi: 10.1136/vr.104217. PubMed PMID: 28866610; PubMed Central PMCID: PMCPMC5738589.

4. Khao Sam Yot Town Municipality, Lopburi, Thailand. Dog population report 2019-2022. 2022.
