# Supplementary material for: Optimizing dog population control strategies in Thailand using mathematical and economic modeling
Source: PLoS Negl Trop Dis. 2025 Jul 3;19(7):e0013202. doi: 10.1371/journal.pntd.0013202 (PMC12225835; doi:10.1371/journal.pntd.0013202)
Supplement: S2 Text — (DOCX) [file pntd.0013202.s002.docx]

**Optimizing dog population control strategies in Thailand using mathematical and economic modeling**

**Supporting Information S2 Text: Model fitting and related information**

# **1. Model fitting result (Markov Chain Monte Carlo, MCMC fitting)**

1.1) The Bayesian framework

The Bayesian framework is a probabilistic model used for statistical inference. It provides a mathematical way to update the probability of a hypothesis as more evidence or information becomes available. The framework is based on Bayes' theorem, which relates the conditional and marginal probabilities of random events.

Bayes' theorem is the foundation of the Bayesian framework [1]. It is expressed as:

$$p\left( H | E \right)=\frac{p\left( E \right)p(E|H)}{p(E)}$$

where:

p(H∣E) is the posterior probability: the probability of the hypothesis H given the evidence E.

p(E∣H) is the likelihood: the probability of the evidence E given that the hypothesis H is true.

p(H) is the prior probability: the initial probability of the hypothesis H before observing the evidence E.

p(E) is the marginal likelihood or evidence: the total probability of the evidence under all possible hypotheses.

In summary, the MCMC method is a versatile and robust approach to model fitting. Its ability to handle complex models, flexibility in dealing with data distribution assumptions, effectiveness in Bayesian analysis, and efficiency in exploring high-dimensional spaces make it an indispensable tool for statisticians and researchers.

1.2) Prior distribution

A uniform distribution was selected as the prior distribution for all parameter values, due to the limited information available on these parameters. The initial minimum and maximum values were determined based on assumptions and a previous study [2] and then refined through an iterative model fitting process.

1.3) Likelihood function

We defined the likelihood as the product of likelihood terms for each data point. The data arises from the total number of dog population that was from the survey between 2019 to 2022. These are linked to the summation of expected values via a Poisson distribution as the following equation.

$$LL= \sum_{t=1}^{i} log(\frac{e^{-\lambda_{i}}\lambda_{i}^{x_{i}}}{x_{i}!})$$

Where:

LL is the log-likelihood.

x_i_​​ represents the observed data points at time *i*.

λ_i_​ represents the mean parameters (the number of dog population) at time *i*.

1.4) Model fitting

Multiple Markov chains were run for a total of 10,000 iterations with the chosen burn-in period of 500 time-steps to guarantee that the samples were not influenced by the initial values. The diagnostic checks were performed to ensure that your MCMC chains mixed well and converged.

**Estimated death rates**

| **Parameter** | **Description** | **psf** | **MAP** | **2.5%** | **Median** | **97.5%** |
| --- | --- | --- | --- | --- | --- | --- |
| Dpup | Free-roaming puppy death rate | 1.005 | 0.001 | 0.0006 | 0.0009 | 0.0011 |
| DAE | Free-roaming adult and elderly death rate | 1.006 | 0.001 | 0.0007 | 0.0009 | 0.0011 |

Convergence, Gelman–Rubin multivariate psrf: 1.005

**Trace plots and posterior distributions of death rates**


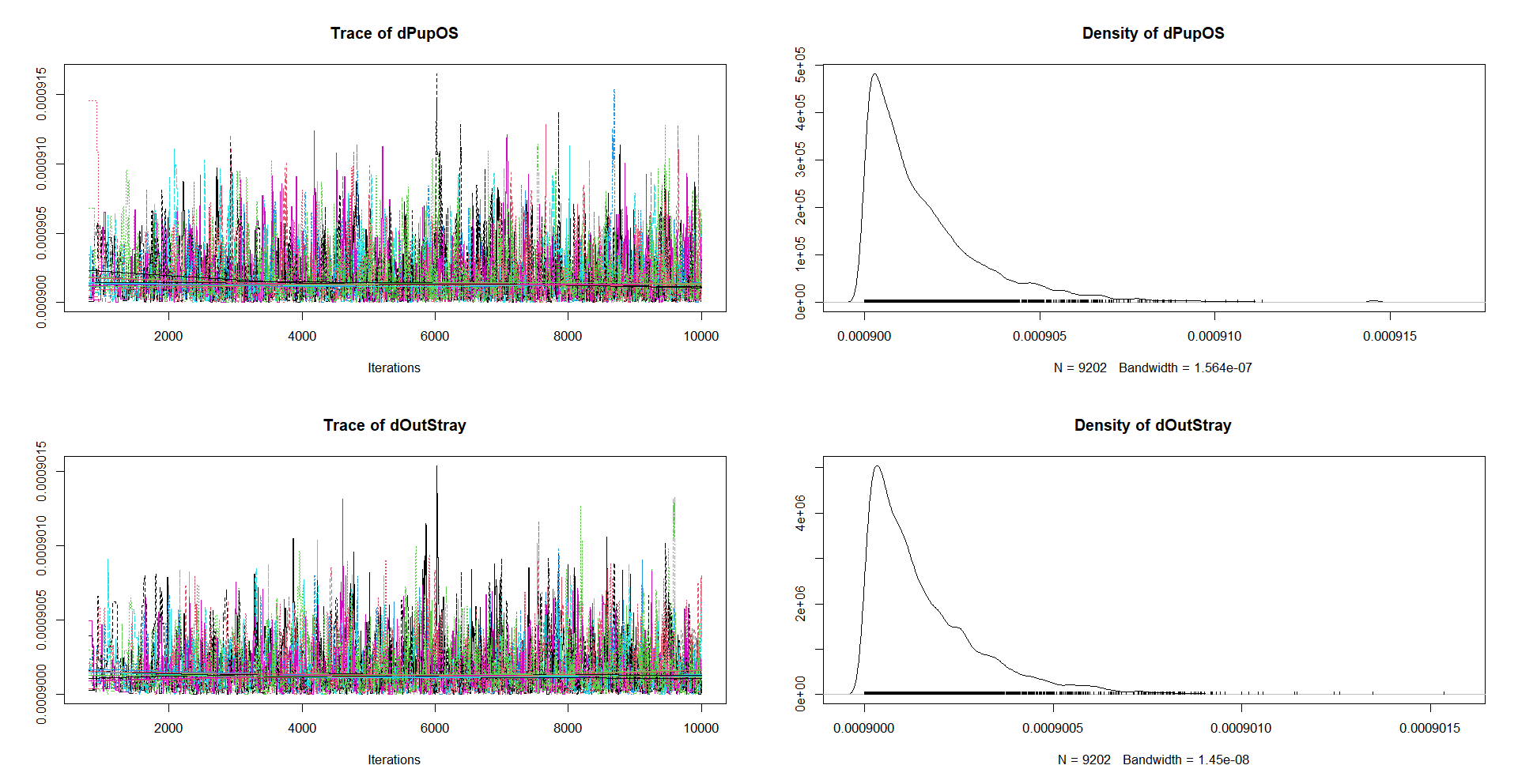


# **2. Data available for this study**

2.1) The number of dogs divided by vaccination and sterilization program in Khao Sam Yot, Lopburi provinces

| Year | Total dogs | Vaccinated dogs | Sterilized dogs |
| --- | --- | --- | --- |
| 2019 | 5943 | 4160.1 | 1333 |
| 2020 | 5885 | 5296.5 | 1821 |
| 2021 | 5493 | 4943.7 | 1756 |
| 2022 | 5202 | 4161.6 | 1040 |

2.2) The number of dogs divided by type in Khao Sam Yot, Lopburi province

| Year | Owned dogs | Stray dogs |
| --- | --- | --- |
| 2019 | 4820 | 1123 |
| 2020 | 4480 | 1405 |
| 2021 | 4171 | 1322 |
| 2022 | 4075 | 1127 |

**References**

1. Hartig F, Minunno F, Paul S, Cameron D, Ott T, M. P. General-Purpose MCMC and SMC Samplers and Tools for Bayesian Statistics 2023 [16 September 2023]. Available from: <https://github.com/florianhartig/BayesianTools>.

2. Komol P, Sommanosak S, Jaroensrisuwat P, Wiratsudakul A, Leelahapongsathon K. The Spread of Rabies Among Dogs in Pranburi District, Thailand: A Metapopulation Modeling Approach. Front Vet Sci. 2020;7:570504. Epub 2020/12/18. doi: 10.3389/fvets.2020.570504. PubMed PMID: 33330692; PubMed Central PMCID: PMCPMC7710610.
