# Supplementary material for: Optimizing dog population control strategies in Thailand using mathematical and economic modeling
Source: PLoS Negl Trop Dis. 2025 Jul 3;19(7):e0013202. doi: 10.1371/journal.pntd.0013202 (PMC12225835; doi:10.1371/journal.pntd.0013202)
Supplement: S3 Text — (DOCX) [file pntd.0013202.s003.docx]

**Optimizing dog population control strategies in Thailand using mathematical and economic modeling**

**Supporting Information S3 Cost evaluation and related information**

# **1. Vaccination coverage and R_t_**


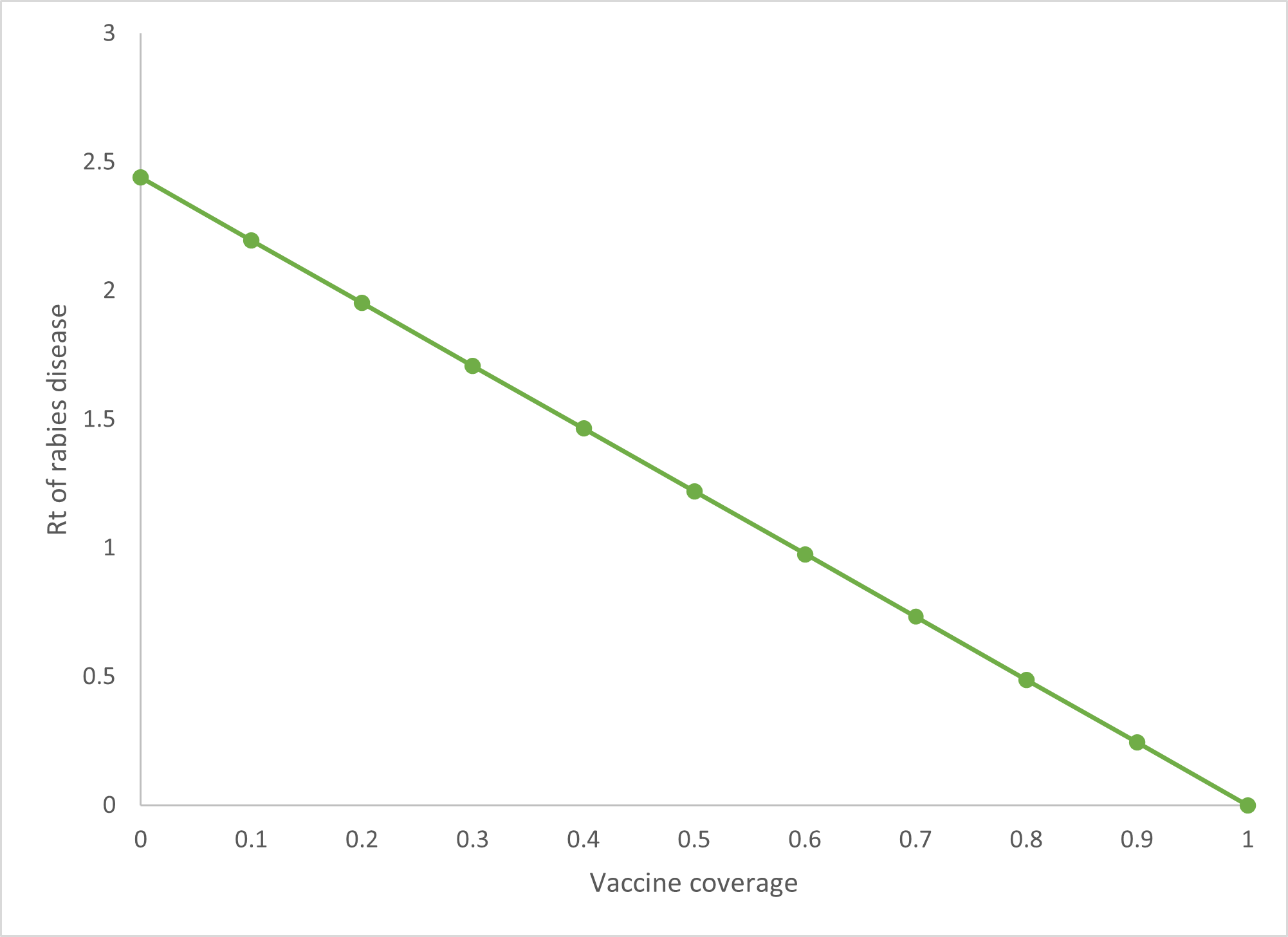


The 70% coverage is considered in the cost-effectiveness evaluation based on Thailand and WHO guidelines.

# **2. An incremental cost-effectiveness ratio (ICER) calculation**

$$ICER=\frac{{Five year cost}_{\left( \mathrm{scenario} \right)}- {Five year cost}_{(current intervention)}}{\#{Dog in 2027}_{(scenario)}- {\#Dog in 2027}_{(current intervention)}}$$

# **3. Cost estimation**

3.1) Sterilization costs by strategies and by year


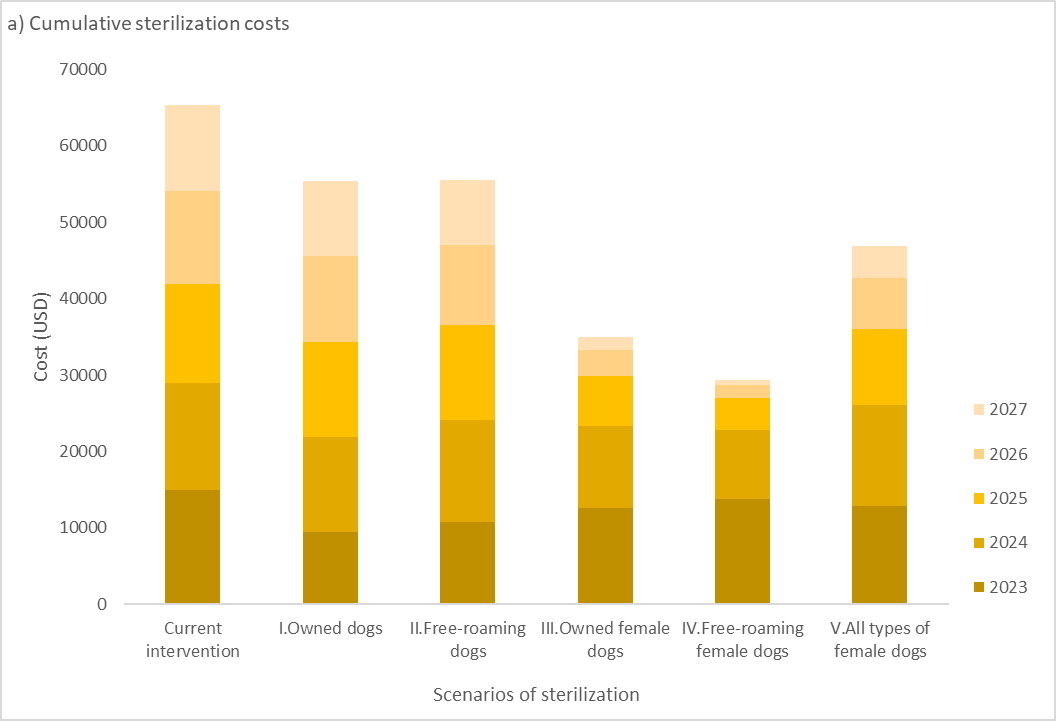


3.2) Rabies vaccination costs by strategies and by year


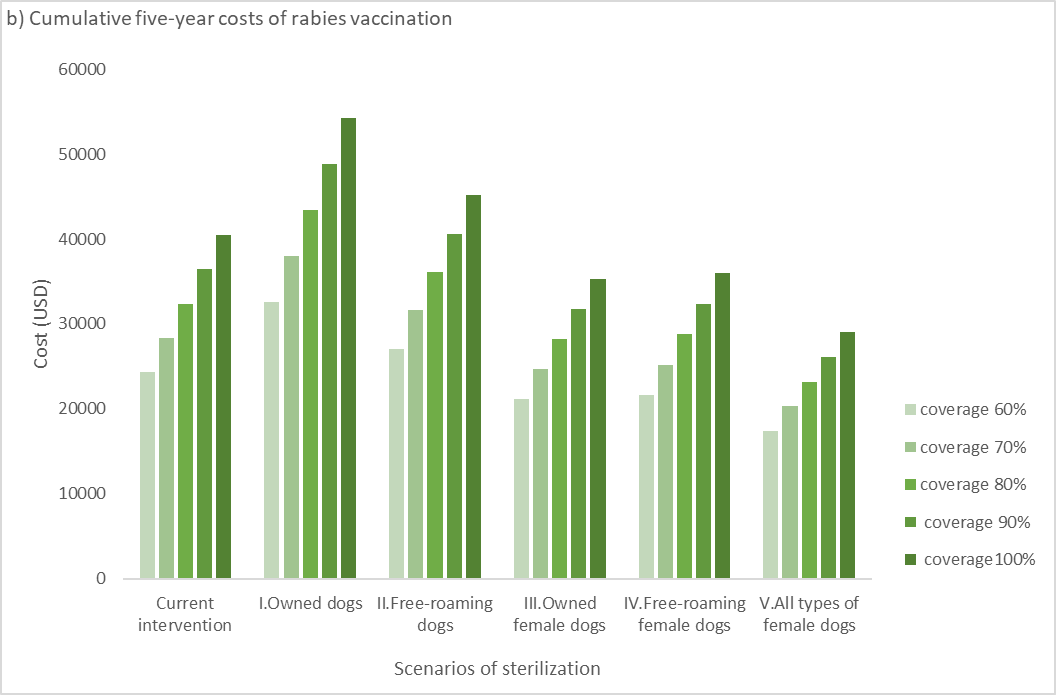


3.3) Average sterilization cost per dog by strategies and by year


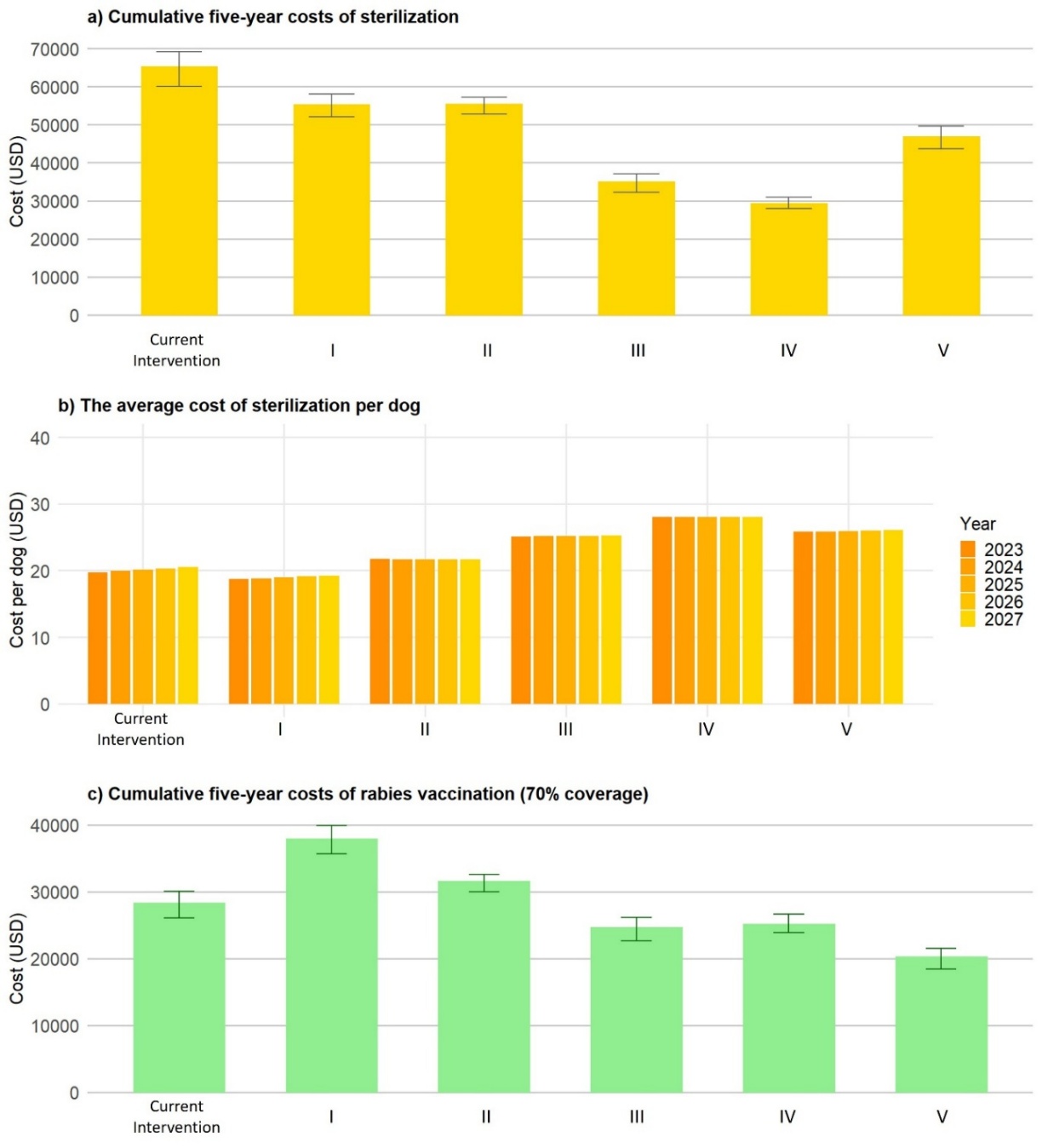


The scenarios are sterilization programs of, I) Owned Dogs, II) Free-Roaming Dogs, III) Owned Female Dogs, IV) Free-Roaming Female Dogs, and V) All Female Dogs.
