# Supplementary material for: Optimizing dog population control strategies in Thailand using mathematical and economic modeling
Source: PLoS Negl Trop Dis. 2025 Jul 3;19(7):e0013202. doi: 10.1371/journal.pntd.0013202 (PMC12225835; doi:10.1371/journal.pntd.0013202)
Supplement: S1 Table — (DOCX) [file pntd.0013202.s004.docx]

**Optimizing dog population control strategies in Thailand using mathematical and economic modeling**

**Supporting information S4 Table: The estimated number of dogs in the five-year scenarios divided by sex and sterilization status.**

| **Scenario of sterilization (st)** | **2023** | **2024** | **2025** | **2026** | **2027** |
| --- | --- | --- | --- | --- | --- |
| **I. Owned dogs** |  |  |  |  |  |
| Total_male.indoor | 917 | 715 | 557 | 435 | 339 |
| total_male.outdoor | 1025 | 911 | 811 | 722 | 643 |
| total_male.stray | 740 | 970 | 1276 | 1678 | 2208 |
| total_female.indoor | 691 | 539 | 420 | 328 | 256 |
| total_female.outdoor | 773 | 687 | 612 | 545 | 485 |
| total_female.stray | 558 | 732 | 963 | 1266 | 1666 |
| total_st.male.indoor | 147 | 186 | 174 | 148 | 120 |
| total_st.male.outdoor | 141 | 191 | 196 | 184 | 167 |
| total_st.male.stray | 0 | 0 | 0 | 0 | 0 |
| total_st.female.indoor | 111 | 140 | 132 | 112 | 91 |
| total_st.female.outdoor | 107 | 144 | 148 | 139 | 126 |
| total_st.female.stray | 0 | 0 | 0 | 0 | 0 |
|  |  |  |  |  |  |
| **II. Free-roaming dogs** |  |  |  |  |  |
| total_male.indoor | 1290 | 1449 | 1631 | 1835 | 2066 |
| total_male.outdoor | 915 | 721 | 569 | 449 | 354 |
| total_male.stray | 444 | 344 | 269 | 210 | 165 |
| total_female.indoor | 973 | 1093 | 1230 | 1385 | 1558 |
| total_female.outdoor | 691 | 544 | 429 | 338 | 267 |
| total_female.stray | 343 | 268 | 210 | 164 | 129 |
| total_st.male.indoor | 0 | 0 | 0 | 0 | 0 |
| total_st.male.outdoor | 180 | 224 | 209 | 177 | 144 |
| total_st.male.stray | 99 | 122 | 112 | 94 | 76 |
| total_st.female.indoor | 0 | 0 | 0 | 0 | 0 |
| total_st.female.outdoor | 136 | 169 | 158 | 134 | 109 |
| total_st.female.stray | 83 | 99 | 90 | 76 | 61 |
|  |  |  |  |  |  |
| **III. Owned female dogs** |  |  |  |  |  |
| total_male.indoor | 804 | 460 | 237 | 114 | 52 |
| total_male.outdoor | 830 | 488 | 258 | 128 | 60 |
| total_male.stray | 743 | 980 | 1296 | 1713 | 2266 |
| total_female.indoor | 350 | 131 | 48 | 18 | 7 |
| total_female.outdoor | 381 | 158 | 66 | 27 | 11 |
| total_female.stray | 561 | 740 | 978 | 1293 | 1709 |
| total_st.male.indoor | 0 | 0 | 0 | 0 | 0 |
| total_st.male.outdoor | 0 | 0 | 0 | 0 | 0 |
| total_st.male.stray | 0 | 0 | 0 | 0 | 0 |
| total_st.female.indoor | 256 | 216 | 129 | 68 | 33 |
| total_st.female.outdoor | 244 | 209 | 128 | 68 | 34 |
| total_st.female.stray | 0 | 0 | 0 | 0 | 0 |
|  |  |  |  |  |  |
| **IV. Free-roaming female dogs** |  |  |  |  |  |
| total_male.indoor | 1285 | 1438 | 1611 | 1806 | 2025 |
| total_male.outdoor | 695 | 332 | 142 | 57 | 22 |
| total_male.stray | 346 | 165 | 70 | 28 | 11 |
| total_female.indoor | 970 | 1085 | 1216 | 1363 | 1528 |
| total_female.outdoor | 218 | 49 | 11 | 2 | 1 |
| total_female.stray | 108 | 24 | 5 | 1 | 0 |
| total_st.male.indoor | 0 | 0 | 0 | 0 | 0 |
| total_st.male.outdoor | 0 | 0 | 0 | 0 | 0 |
| total_st.male.stray | 0 | 0 | 0 | 0 | 0 |
| total_st.female.indoor | 0 | 0 | 0 | 0 | 0 |
| total_st.female.outdoor | 307 | 202 | 96 | 40 | 16 |
| total_st.female.stray | 188 | 118 | 54 | 22 | 8 |
|  |  |  |  |  |  |
| **V. All types of female dogs** |  |  |  |  |  |
| total_male.indoor | 1008 | 754 | 518 | 339 | 215 |
| total_male.outdoor | 982 | 717 | 485 | 314 | 199 |
| total_male.stray | 490 | 357 | 241 | 156 | 99 |
| total_female.indoor | 542 | 322 | 191 | 113 | 67 |
| total_female.outdoor | 551 | 338 | 208 | 128 | 78 |
| total_female.stray | 275 | 168 | 103 | 63 | 39 |
| total_st.male.indoor | 0 | 0 | 0 | 0 | 0 |
| total_st.male.outdoor | 180 | 224 | 209 | 177 | 144 |
| total_st.male.stray | 99 | 122 | 112 | 94 | 76 |
| total_st.female.indoor | 0 | 0 | 0 | 0 | 0 |
| total_st.female.outdoor | 136 | 169 | 158 | 134 | 109 |
| total_st.female.stray | 83 | 99 | 90 | 76 | 61 |
